# N6-Methyladenosine-Related lncRNAs Are Anticipated Biomarkers for Sarcoma Patients

**DOI:** 10.1155/2022/1093805

**Published:** 2022-05-27

**Authors:** Xiaohe Zhang, Hongbo Li, Liuzhi Zhou, Di Wu, Shixiang Zhou, Yao Yang, Yabin Hu

**Affiliations:** ^1^Department of Orthopedics, The Second Hospital of Nanjing, Nanjing University of Chinese Medicine, Nanjing, Jiangsu 210003, China; ^2^Department of Musculoskeletal Oncology, The First Affiliated Hospital of Sun Yat-sen University, Guangzhou, Guangdong 510080, China; ^3^Department of Surgery, The Second Affiliated Hospital, Zhejiang University School of Medicine, Hangzhou, Zhejiang 310009, China; ^4^Department of Neurology, Eastern Theater General Hospital Qinhuai District Medical Area, Nanjing, Jiangsu 210000, China

## Abstract

**Background:**

Soft tissue sarcomas (STSs) are rare tumors and occur at any site in the body. Our goal was to identify a putative molecular mechanism for N6-methyladenosine (m6A) lncRNA alteration and to develop predictive biomarkers for sarcoma.

**Methods:**

The lncRNA levels were obtained from TCGA datasets. Pearson correlation analysis was used to select all the lncRNAs that are connected to m6A. An m6A-related lncRNA model was built using LASSO Cox regression. To assess the prognostic efficiency of the model and potential lncRNAs, we performed univariate survival analysis and receiver operating characteristic (ROC) analysis. We also performed enrichment analysis to evaluate the roles of the potential genes. Finally, quantitative real-time polymerase chain reaction (qRT-PCR) was utilized to confirm m6A-related lncRNA expression in tissues.

**Results:**

Following Pearson correlation analysis on TCGA datasets, we identified 78 m6A-related lncRNAs. Next, we used LASSO Cox regression analysis and identified 13 m6A-related lncRNAs as prognostic lncRNAs. After calculating risk scores, sarcoma patients were divided into high- and low-risk groups depending on the median of risk scores. We also found that these lncRNAs were immune associated via enrichment analysis.

**Conclusions:**

Here, we found that SNHG1, FIRRE, and YEATS2-AS1 could serve as biomarkers to predict overall survival of sarcoma patients, which provides a new insight into treatment of STS.

## 1. Introduction

Soft tissue sarcomas (STSs) occupy less than 1% of all tumors. STSs can occur in any age, with an increasing rate in older patients. There is no significant difference in the incidence of this tumor between males and females [1, 2]. To date, surgical excision with wide margins remains the major treatment for STSs. In addition to surgery, radiotherapy has been consistently used, although it is still debated which parameters should be used to identify the tumors that are more likely to benefit from radiotherapy [3].

RNA splicing, stability, export, and translation are all affected by N6-methyladenosine (m6A) alteration [4, 5]. The m6A modification was regarded as an avertible and active RNA epigenetic process, known as “writers,” “readers,” and “erasers” [6]. The m6A alteration is crucial in regulating oncogenesis and tumor growth in different types of tumors [7, 8]. For instance, METTL14 exerts its oncogenic role by regulating m6A modification of its mRNA targets in acute myeloid leukemia cells [7]. YTHDF2 inhibition promotes cell growth by reducing the m6A modification-induced degradation of EGFR mRNA in hepatocellular carcinoma (HCC) cells [8]. Previous studies have used m6A-related lncRNA prognostic models to predict the prognosis of individuals with lower-grade gliomas [9]. The immune system-associated lncRNAs have considerable impacts on gene expression of articulation, including immune activation and immunosuppression [10]. When TUC339, an HCC-derived exosomal lncRNA, was used to control the polarization of M1/M2 macrophages, it affected the complex immunological microenvironment between tumor and immune cells [11]. The lncRNA GATA3-AS1 boosted neoplasm invasion and immune evasion in triple-negative breast cancer by maintaining PD-L1 protein and degrading GATA3 protein [12]. Sarcoma and m6A-related lncRNAs are yet to be studied in detail.

13 m6A-related lncRNAs were found, and their predictive potentials in sarcoma patients were further examined. In addition, we developed a risk model to forecast the survival of sarcoma patients. Our final step was to evaluate m6A-related lncRNA expression in tumor tissues and nontumor tissues to confirm the results of bioinformatics analysis.

## 2. Materials and Methods

### 2.1. Data Acquisition and Processing

Gene expression information in normal or sarcoma tissues was retrieved from TCGA (https://portal.gdc.cancer.gov/) in March 2021. All data was standardized with the package “limma.” TCGA-sarcoma sewing data for the RNA (FPKM value) were acquired from the “Genomic Data Commons” and collected for further analysis as a training/testing set. The gene profile expression was assessed by FPKM and standardized log^2^ transformation. Furthermore, m6A-related expression levels of 13 genes are downloaded from published publications (AC004076.2, AC022973.4, YEATS2-AS1, AP000692.1, ITGA6-AS1, AL139289.1, FIRRE, AC008735.2, AL031985.3, AC026271.3, SNHG1, LINC02447, and AC087645.2). lncRNAs were annotated by GENECODE. Finally, we obtained 14,143 TCGA lncRNAs based on the information about the annotations and their ensemble IDs.

### 2.2. Analyzing the Relationship between lncRNAs and m6A-Related Genes

The Pearson correlation coefficient was utilized to determine the relationship between the 13 m6A-related genes and 14,143 lncRNAs. We identified lncRNAs with a Pearson correlation coefficient (PCC) > 0.5 and *P* value < 0.001 as m6A-related lncRNAs.

### 2.3. Univariate Cox Regression Analysis and Consensus Cluster of m6A Regulators

In order to differentiate the prognostic lncRNAs, the univariate Cox assessment was conducted. We have divided sarcoma patients into two cluster groups by *k*-means clustering depending on m6A-associated lncRNAs. The ConsensusClusterPlus R program was used for the analysis of clusters.

### 2.4. m6A Patterns: Immune Cell Infiltration Analysis

Estimate, immune, and stromal scores were obtained depending on the ESTIMATE program to evaluate the proportion of immune cell between the two subgroups of the study population. Software CIBERSORT was utilized to analyze the distribution of 22 immune cell subtypes derived from TCGA-sarcoma samples in order to determine the differences between two clusters.

### 2.5. GSEA Pathways

According to calculated scores of the m6A-related lncRNAs, we divided the sarcoma samples into cluster 1 and cluster 2. GSEA was utilized to investigate the gene enrichment pathway in each cluster.

### 2.6. LASSO Cox Analysis for Predictive Model Design

LASSO Cox regression was utilized to develop an m6A-related lncRNA prognostic model of sarcoma patients using glmnet R package. Risk score was calculated according to the formula: risk score = expression_lncRNA1_ × coefficient_lncRNA1_ + expression_lncRNA2_ × coefficient_lncRNA2_ + ⋯e xpression_lncRNA*n*_ × coefficient_lncRNA*n*_. The patients were separated into two groups: high-risk and low-risk groups based on their median risk score. Sarcoma's Kaplan-Meier OS curves were utilized to assess the accessibility of the prognostic model. Besides, the receiver operator characteristic (ROC) curve was also employed to evaluate the predictive accuracy of the m6A-lncRNA signature.

### 2.7. Real-Time Polymerase Chain Reaction (qRT-PCR)

We have completely acquired 38 normal and tumor samples from sarcoma patients who got surgical therapies in the Department of Musculoskeletal Oncology, Sun Yat-sen University's First Affiliated Hospital, from 2015 to 2019. Tissue was frozen promptly and preserved at -80°C. Experiments were authorized by the Medical Ethics Committee of Sun Yat-sen University's First Affiliated Hospital. The sample was handled in accordance with approved guidelines. Each participating patient has signed informed consents. We measured the expression of m6A-related lncRNAs, with GAPDH as an endogenous control, after extracting total RNA from clinical sarcoma samples with RNA Trizol reagent. Primer sequence orientations (5′→3′) are as follows: AL031985.3 forward AGGAAATGACCCGAACTGCC and reverse ATTGAACTGAGCGGGGCTTT; SNHG1 forward CAATGTTCAGCCCACAAGAGC and reverse CCCTTTGAGCCAAGCAGGTT; FIRRE forward TGAAAGGGAATCCTGACGCC and reverse TGCCTAGCTCTGACAATGGC; LINC02447 forward ACGTGGGTTTCCGTATCCTC and reverse TCTGTTCTCCTCTGTTGTTTCAGG; and YEATS2-AS1 forward AGCCGTTTGTTCGTATCGCT and reverse ATTCCGTGTTCCTTTCCCGT.

### 2.8. Statistical Analysis

Analysis was performed with R.3.3.3 (R Foundation for Statistical Computing, Vienna, Austria), including the survival analysis Kaplan-Meier and the Cox multivariate and univariate analysis. Pearson correlation analysis was used to investigate the relationship between the risk score and activated immune cells including CD8^+^ T cells, M0 macrophages, and M1 macrophages. All statistical *P* values were bilateral, and *P* < 0.05 was considered statistically significant.

## 3. Results

### 3.1. Identification of m6A-Related lncRNAs in STS Patients

**Figure 1:**
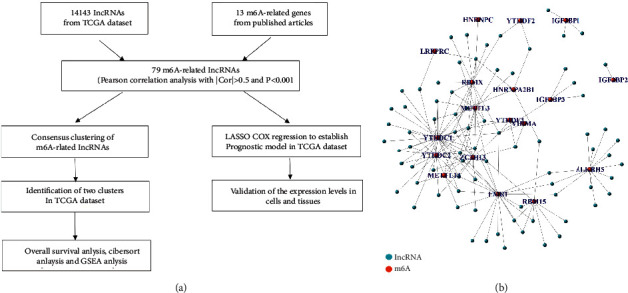
(a) Study flowchart. (b) The network of m6A-related genes and lncRNAs in sarcoma.

A total of 14,143 lncRNAs were identified in TCGA dataset, and the matrixes of 13 m6A-related gene expression were derived from the same dataset. We identified lncRNAs with a value associated to one or many m6A-related genes and defined them as m6A-related lncRNAs (|Pearson *R*| > 0.5 and *P* < 0.001). 78 lncRNAs were identified. Univariate Cox regression analysis was performed for the screening of prognosis-specific m6A-related lncRNAs (*P* value < 0.05). Finally, 13 m6A-related lncRNAs were found from TCGA dataset. The workflow is shown in Figure 1(a). A network of the m6A-related genes and lncRNAs is shown in Figure 1(b).

### 3.2. Consensus Clustering of m6A-Related lncRNAs in Two Clusters

**Figure 2:**
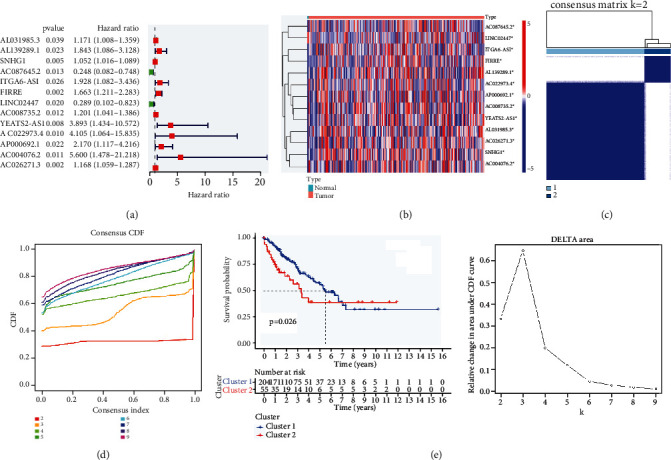
Consensus clustering of m6A-related lncRNAs. (a) Forest plot of prognostic ability of the m6A-related lncRNAs. (b) Heat map of the expression levels of AC004076.2, AC022973.4, YEATS2-AS1, AP000692.1, ITGA6-AS1, AL139289.1, FIRRE, AC008735.2, AL031985.3, AC026271.3, SNHG1, LINC02447, and AC087645.2 between normal and tumor tissues. (c) Consensus clustering matrix for *k* = 2. (d) Consensus clustering cumulative distribution function (CDF) and relative change in area under CDF curve for *k* = 2. (e) Kaplan-Meier curves of OS for two clusters in TCGA.

Univariate Cox regression analysis was used to evaluate the prognostic roles of 13 m6A-related lncRNAs. The forest plot shows that AC004076.2, AC022973.4, YEATS2-AS1, AP000692.1, ITGA6-AS1, AL139289.1, FIRRE, AC008735.2, AL031985.3, AC026271.3, and SNHG1 are risk factors with HR (hazard ratio) > 1, while LINC02447 and AC087645.2 are protective factors with HR < 1 in sarcoma patients (Figure 2(a)). The heat map shows that only LINC02447 expression decreased in the tumor tissue, whereas the expression of the other lncRNAs increased in the tumor tissue (Figure 2(b)).

Derived from the ConsensusClusterPlus R package, TCGA-sarcoma cohort was divided into cluster 1 and cluster 2 by consensus expression of m6A regulators. The optimal number of clusters (*k* = 2) was confirmed with optimal clustering stability *k* = 2–9 (Figures 2(c) and 2(d)). The Kaplan-Meier method was used to calculate the overall survival among clusters. The survival rate in cluster 1 is significantly higher than that in cluster 2 (Figure 2(e)).

### 3.3. Immune Patterns in Sarcoma Patients

**Figure 3:**
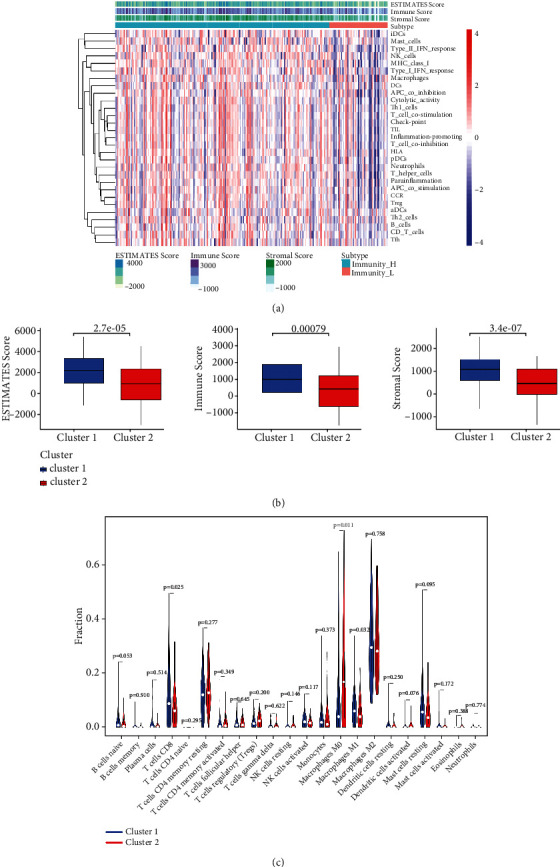
Immune characteristics among three m6A patterns. (a) The heat map of association of m6A regulators from 2 clusters with the estimate, stromal, and immune scores using ESTIMATE algorithm. (b) Estimate score, immune score, and stromal score in cluster 1 and cluster 2. (c) Differences in the levels of infiltration of the 22 immune cells in m6A-related lncRNAs in cluster 1 and cluster 2.

The estimate, stromal, and immune scores of sarcoma patients are calculated by the ESTIMATE R package. The heat map of m6A-related lncRNA expression and the three scores are shown in Figure 3(a). We found that such m6A-related lncRNAs presented a reverse trend with stromal, immune, and estimate scores, implying that the m6A-related lncRNA may undertake a vital role in the tumor immune microenvironment. Cluster 1 has significantly higher immune, estimate, and stromal scores than cluster 2 (Figure 3(b)). We used the CIBERSORT algorithm to analyze the abundance of 22 different immune cells in the two clusters. Cluster 1 displayed a greater proportion of CD8^+^ T cells and M1 macrophages than cluster 2 (Figure 3(c)). On the contrary, the lower proportion of M0 macrophages was found in cluster 1. These results revealed that m6A-related patterns may influence the response to immunotherapy via adjusting the expression of specific immune cell types.

### 3.4. Pathway Enrichment Analysis and Gene Set Enrichment Analysis (GSEA)

**Figure 4:**
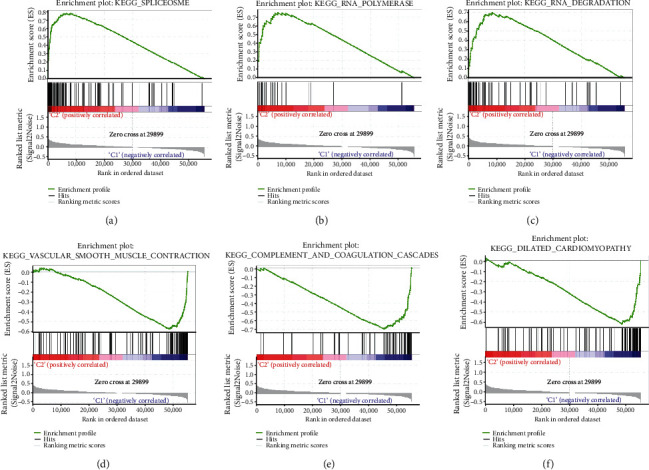
Gene set enrichment analysis (GSEA) with cluster 1 and cluster 2. (a–c) The top 3 pathways enriched in cluster 2 included spliceosome, RNA polymerase, and RNA degradation. (d–f) The top 3 pathways enriched in cluster 1 included vascular smooth muscle contraction, complement and coagulation cascades, and dilated cardiomyopathy.

By GSEA of TCGA cohort, the differentially expressed genes of cluster 2 were mainly enriched in the spliceosome, RNA polymerase, and RNA degradation pathways (Figures 4(a)–4(c)). In cluster 1, the differentially expressed genes had close association with vascular smooth muscle contraction, complement and coagulation cascades, and dilated cardiomyopathy (Figures 4(d)–4(f)).

### 3.5. Construction of the m6A-Related lncRNA-Based Risk Signature in SARC

**Figure 5:**
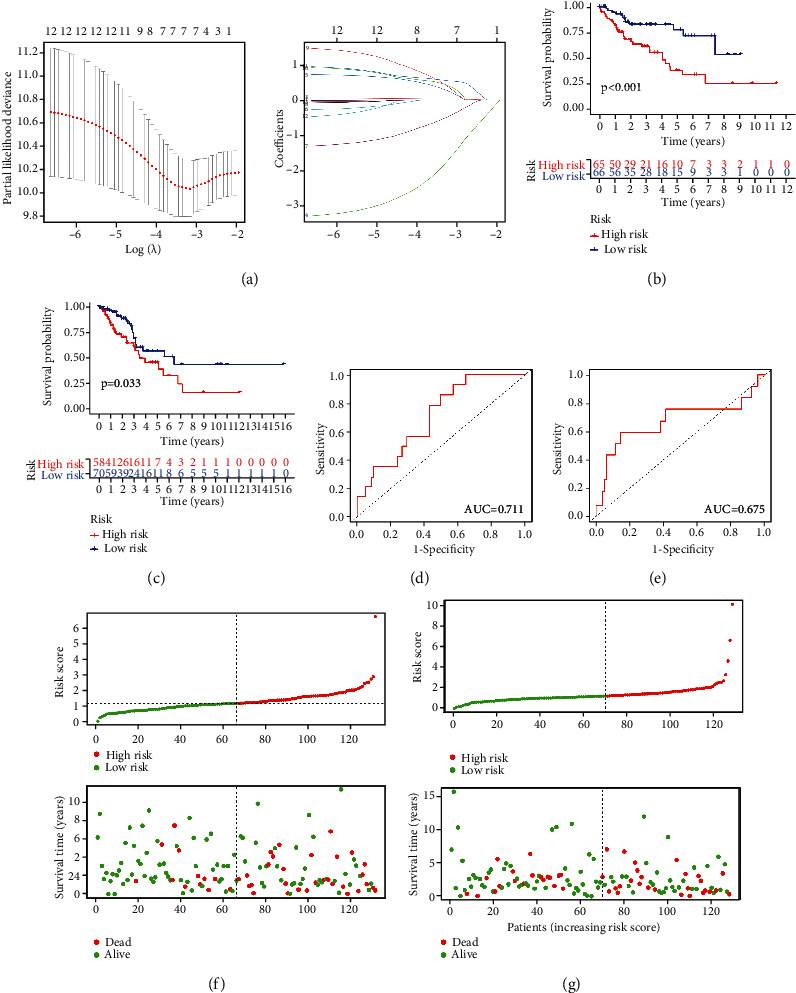
Risk model from m6A-related lncRNAs. (a) LASSO Cox regression analysis of m6A-related lncRNAs. (b, c) Overall survival analysis for patients in high/low risk of train/test set. (d, e) The ROC curve of risk score of train/test set. (f, g) The distributions of risk scores, alive/dead status of train/test set.

A risk model to predict prognosis of sarcoma patients was constructed based on the multivariate Cox regression analysis, and the coefficients are shown in Figure 5(a). After dividing sarcoma patients into two groups depending on the median of risk scores, we analyzed samples with ROC curve and overall survival analysis. Sarcoma patients in the high-risk group displayed significantly shorter overall survival than those in the low-risk group (Figures 5(b) and 5(c)). Additionally, the ROC curve identified the excellent performance of this risk signature in predicting overall survival of sarcoma patients (Figures 5(d) and 5(e)). Patients in the high-risk group had shorter survival time and worse survival status (Figures 5(f) and 5(g)).

### 3.6. Verifying m6A-Related lncRNA Expressions in STS Tissues

**Figure 6:**
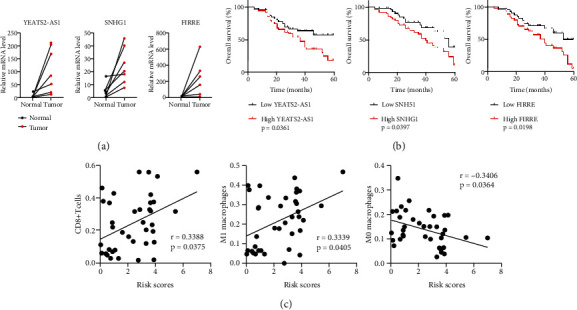
Evaluating expression of m6A-related lncRNAs in STS tissues. (a) Expression of SNHG1, FIRRE, and YEATS2-AS1 in 38 STS specimens and normal specimens. (b) Overall survival curves that showed the association of SNHG1, FIRRE, and YEATS2-AS1 with STS prognosis. (c) The correlations between the risk score and immune cells including CD8^+^ T cells, M1 macrophages, and M0 macrophages. ^∗∗∗^*P* < 0.001.

In order to further determine the expression patterns of these m6A-related lncRNAs, we analyzed their relative expression in normal and STS tissues using qRT-PCR. As shown in Figure 6(a), the SNHG1, FIRRE, and YEATS2-AS1 expression levels were significantly highly expressed in STS tissues compared with normal tissues. Higher expressions of SNHG1, FIRRE, and YEATS2-AS1 were associated with the lower overall survival of patients with STS (Figures 6(b)–6(d)). Thus, these results indicated that SNHG1, FIRRE, and YEATS2-AS1 could serve as prognostic biomarkers in sarcoma. Moreover, the risk score was positively related to activated CD8^+^ T cells and M1 macrophages and negatively related to M0 macrophages (Figure 6(e)).

## 4. Discussion

STSs are rare tumors, occupying less than 1% of all tumors. Despite the advancements in the fields of radiology, pathology, and surgery that have been achieved, the treatment for STSs was still unsatisfied because of local recurrence and/or metastasis. Lacking useful biomarkers was considered as one of the important clinical problems. Because of this, the discovery of biomarkers used to predict the prognosis of STSs could help clinicians provide more effective clinical treatment. lncRNAs are non-protein-coding molecules longer than 200 nucleotides and participate in the activities of many types of tumors. Numerous studies have confirmed lncRNAs regulate cancer cell metastasis, proliferation, and chemotherapeutic drug resistance [13, 14]. Therefore, it is crucial to explore the mechanism function of lncRNAs in sarcoma and its connection with sarcoma prognosis.

According to previous studies, the function of m6A includes methyltransferases, demethylases, and binding proteins [15–17]. However, the role of m6A regulators in sarcoma prognosis is still unclear. In order to investigate the prognostic importance of m6A-related lncRNAs, we analyzed 14,143 lncRNAs from TCGA datasets. We generated two clusters based on TCGA dataset using optimal *k*-means clustering and found a substantial difference in overall survival between the two groups, implying that these m6A-related lncRNAs are closely linked to the prognosis of sarcoma.

The tumor immune microenvironment has been the subject of a growing number of research. Cluster 1 was found to be enriched in vascular smooth muscle contraction, complement and coagulation cascades, and dilated cardiomyopathy pathways, while cluster 2 was found to be enriched in spliceosome, RNA polymerase, and RNA degradation pathways, according to GSEA. Furthermore, the expression of m6A-related lncRNAs was substantially linked with the estimate score, immunological score, and stromal score. Similarly, abundance of M1 macrophages was significantly higher in cluster 1 compared to cluster 2, but that of M0 macrophages was significantly lower. These findings offer a thorough examination of m6A-related lncRNAs, which will aid in the development of customized new therapeutics by determining immunotherapy response.

In TCGA datasets, 13 m6A-related lncRNAs were proven to have predictive significance, and we sought to build a risk model for predicting the overall survival of sarcoma patients. All the patients are divided into two groups: training and testing. Sarcoma patients were separated into low- and high-risk subgroups based on their median risk score, with the high-risk group having worse clinical outcomes in both the train and test sets. With area under the curve (AUC) values > 0.6 in both the train and test sets, our predictive risk signature is accurate. Among these 13 lncRNAs, SNHG1, FIRRE, and YEATS2-AS1 were confirmed to be upregulated in clinical STS specimens and predicted poor overall survival of STS patients. By upregulating miR-376a and downregulating FOXK1 and Snail, SNHG1 has been shown to increase HCC cell viability, invasion, and migration, as well as suppress apoptosis [18]. Furthermore, increased SNHG1 expression enhances bladder cancer cell proliferation, invasion, and autophagy via the miR-493-5p/ATG14/autophagy pathway [19]. By sponging miR-520a-3p and regulating YOD1 [20], lncRNA FIRRE function as a new mediator in gallbladder cancer progression. By boosting CREB-mediated PFKFB4 transcription and expression [21], the highly expressed FIRRE promoted hepatocellular carcinoma cell proliferation and glycolysis. In our research, these lncRNAs were discovered to be tightly linked to immunity. In comparison to prior lncRNA investigations, the 13 lncRNAs discovered in this study are relatively new and have clinical potential. As a result, we expect that our findings will aid in the identification of possible prognostic lncRNAs regulated by m6A and thus provide suggestions for improving sarcoma's dismal prognosis.

A recent study also reveals 13 m6A-related lncRNAs in STS [12]. The previous study demonstrated that cluster 1 had higher abundance of M0 macrophages and activated dendritic cells and lower abundance of CD8^+^ T cells [22]. The authors suggested the positive association of risk score and M0 macrophages. Compared with this study, we found that cluster 1 had higher abundance of CD8^+^ T cells and M1 macrophages and lower abundance of M0 macrophages and revealed the negative association of risk score and M0 macrophages. Moreover, we validated the expression levels of 13 m6A-related lncRNAs in STS specimens and revealed that only SNHG1, FIRRE, and YEATS2-AS1 showed upregulation in STS.

In conclusion, we discovered a signature of 13 m6A-related lncRNAs that might predict prognosis of patients with sarcoma. Three of them including SNHG1, FIRRE, and YEATS2-AS1 were confirmed to be upregulated in clinical STS specimens and predicted poor overall survival of STS patients based on our experimental data. The m6A-related lncRNAs could potentially serve as predictive biomarkers and guide therapeutic treatment methods for sarcoma patients.

## Data Availability

Publicly available datasets were analyzed in this study. Data can be found here: https://portal.gdc.cancer.gov/. All of the authors are responsible for the data.

## Abbreviations

STS: Soft tissue sarcoma
TCGA: The Cancer Genome Atlas
TNM: Tumor-node-metastasis
HR: Hazard ratio
GSEA: Gene set enrichment analysis
ROC: Receiver operating characteristic.
